# Insights on the Neuromagnetic Representation of Temporal Asymmetry in Human Auditory Cortex

**DOI:** 10.1371/journal.pone.0153947

**Published:** 2016-04-20

**Authors:** Alejandro Tabas, Anita Siebert, Selma Supek, Daniel Pressnitzer, Emili Balaguer-Ballester, André Rupp

**Affiliations:** 1 Faculty of Science and Technology, Bournemouth University, Bournemouth, England, United Kingdom; 2 Institute of Pharmacology and Toxicology, University of Zurich, Zürich, Zürich, Switzerland; 3 Department of Physics, Faculty of Science, University of Zagreb, Zagreb, Croatia; 4 Département d’Études Cognitives, École Normale Supérieure, Paris, France; 5 The Bernstein Center for Computational Neuroscience Heidelberg-Mannheim, Mannheim, Baden-Würtemberg, Germany; 6 Department of Neurology, Heidelberg University, Heidelberg, Baden-Würtemberg, Germany; Duke University, UNITED STATES

## Abstract

Communication sounds are typically asymmetric in time and human listeners are highly sensitive to this short-term temporal asymmetry. Nevertheless, causal neurophysiological correlates of auditory perceptual asymmetry remain largely elusive to our current analyses and models. Auditory modelling and animal electrophysiological recordings suggest that perceptual asymmetry results from the presence of multiple time scales of temporal integration, central to the auditory periphery. To test this hypothesis we recorded auditory evoked fields (AEF) elicited by asymmetric sounds in humans. We found a strong correlation between perceived tonal salience of ramped and damped sinusoids and the AEFs, as quantified by the amplitude of the N100m dynamics. The N100m amplitude increased with stimulus half-life time, showing a maximum difference between the ramped and damped stimulus for a modulation half-life time of 4 ms which is greatly reduced at 0.5 ms and 32 ms. This behaviour of the N100m closely parallels psychophysical data in a manner that: i) longer half-life times are associated with a stronger tonal percept, and ii) perceptual differences between damped and ramped are maximal at 4 ms half-life time. Interestingly, differences in evoked fields were significantly stronger in the right hemisphere, indicating some degree of hemispheric specialisation. Furthermore, the N100m magnitude was successfully explained by a pitch perception model using multiple scales of temporal integration of auditory nerve activity patterns. This striking correlation between AEFs, perception, and model predictions suggests that the physiological mechanisms involved in the processing of pitch evoked by temporal asymmetric sounds are reflected in the N100m.

## Introduction

Waveforms of sound sources like speech and music are typically asymmetric in time. The term *temporal asymmetry* [1] has been used to describe auditory stimuli that display different *attack* (sound onset) and *decay times* (sound offset). For example, striking cymbals produce a rapid attack followed by an exponential decay in the waveform amplitude, whereas bowing the same instrument results in a more gradual attack. Thus, temporal asymmetry influences the timbre of a stimulus considerably [2]. The temporal envelope also contributes substantially to the identification of instruments: when instruments producing sounds of asymmetrically shaped temporal envelopes are played backwards, humans often fail to identify the instrument [3]. Furthermore, it is well known that differences in attack and decay times affect perceptual timing [4–6] and duration [7], pitch [8], and loudness [9].

**Fig 1:**
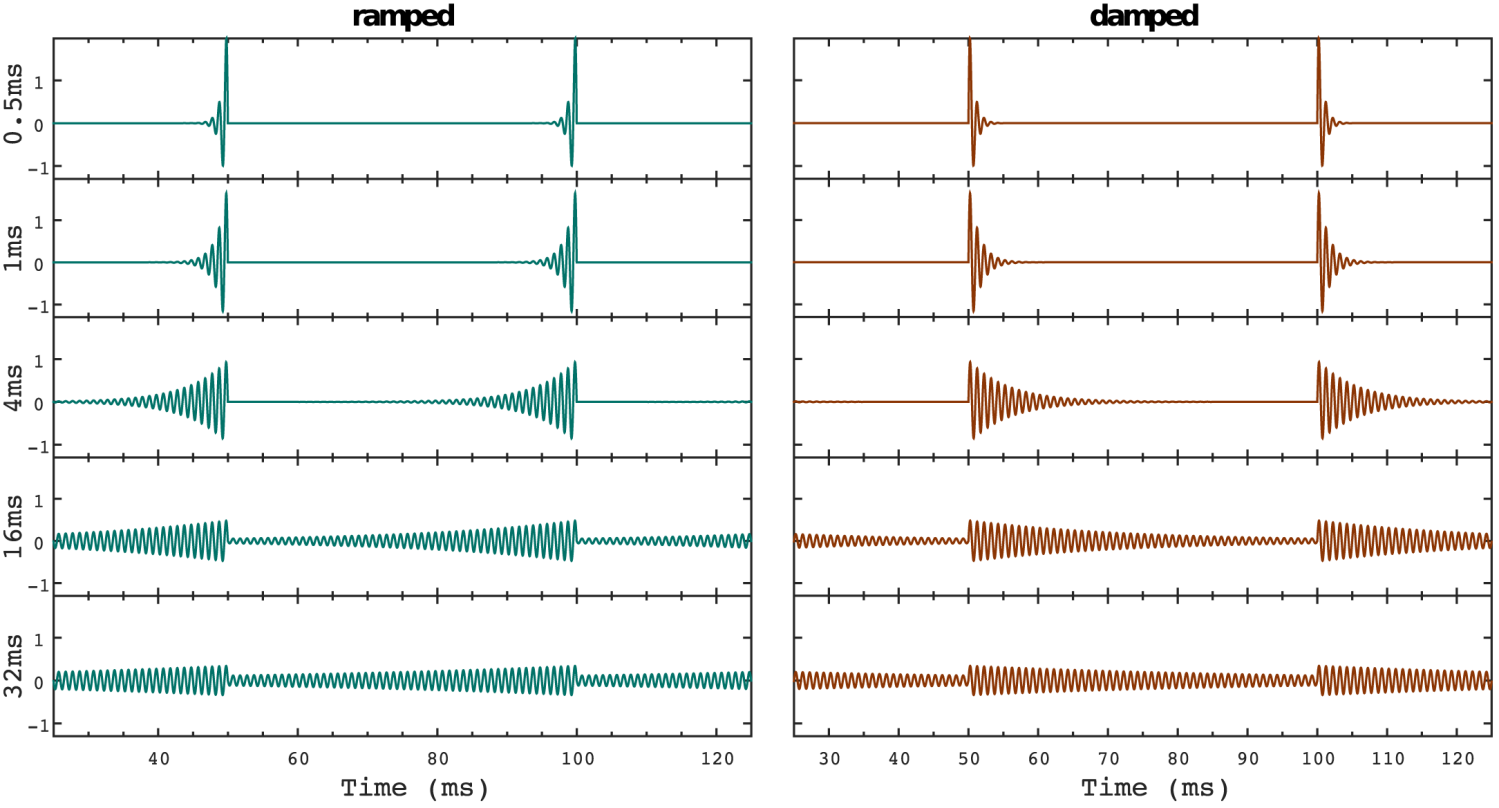
Waveforms of the ramped and damped sinusoids. Ramped (left) and damped (right) sinusoidal waves with half-life times (*T*_1/2_) of 0.5, 1, 4, 16, and 32 ms used in the experiment. Note the two periodicities present in the stimuli corresponding to the carrier (1000 Hz) and the repetition period (20 Hz) of the ramped/damped modulation.

Ramped and damped stimuli [1, 10] enable us to study temporal asymmetry in a systematic fashion. This family of stimuli consists of a sinusoid multiplied either by a periodically rising (*ramped*) or decaying (*damped*) exponential function (see Fig 1). Thus, stimuli present two different periodicities that are perceived simultaneously: the periodicity of the carrier (the fundamental frequency of the pure tone before modulation) and the *envelope*’s one (the periodicity of the modulation pattern). Ramped and damped sinusoids evoke different perceptions: ramped sounds are perceived as continuous tones with the pitch of the carrier, whereas repetitive streams of damped sinusoids are perceived as a drumming sound with a lower pitch salience.

These stimuli pose an interesting problem for the understanding of temporal processing in the auditory system because their long-term Fourier spectra are identical. Hence, models of auditory perception, essentially based on extracting the auditory nerve periodicities on a fixed, and often long, time window (see, e.g., [11–13]) cannot fully explain such perceptual differences.

Here we propose that models incorporating stimulus-dependent adaptive processing of the auditory nerve activity patterns provide an insight into perceptual asymmetry phenomena. One of the earliest models of this kind is the Auditory Image Model (AIM) [14], which simulates the representation of sounds beyond the auditory nerve using an adaptive mechanism for temporal integration called *strobed temporal integration*. This non-linear transform converts the activity pattern of the auditory nerve into the so-called *stabilised auditory image* (SAI), which correlates to the perceived pitch and salience of ramped and damped sounds [15].

More recently, empirical and modelling studies [16–18] offered further evidence of the existence of a stimulus-driven adaptation of the temporal integration window. In a recent model of pitch perception [17] this adaptation was proposed to explain that, while long integration windows are necessary to understand a wide range of perceptual phenomena (e.g. [19–21]), short integration windows are necessary to identify quick variations in the inputs stimuli on the millisecond range [22]. This balance between perceptual integration and resolution was achieved by a top-down modulation process which is sensitive to quick stimulus variations, such as those occurring in temporally asymmetric sounds [17].

Neurophysiological responses to ramped and damped sinusoids have been analysed both in subcortical and in cortical structures (e.g., in ventral cochlear nucleus [23], in inferior colliculus [24] and in primary auditory cortex [25]). Taken together, these electrophysiological studies demonstrate the consistency of the temporal asymmetry in single-unit responses with the perceptual asymmetry. However, those animal studies did not attempt to identify the causal physiological correlate of the perception elicited by damped and ramped sounds in human listeners [24].

In the present work, we combine non-invasive magnetoencephalography (MEG) and perceptual studies in human listeners with pitch perception models in order to better understand the processing of asymmetric sounds. We identified a neuromagnetic representation of auditory perceptual asymmetry in the morphology of the N100m deflection of the auditory evoked fields (AEF). The N100m is a well-known transient neuromagnetic response elicited 100 ms after the tone onset. This deflection arises from multiple sources in auditory cortex, lateral Heschl’s gyrus and planum temporale [26, 27]. As the N100m is sensitive to the intensity [28, 29] and the rise-time of the sound [30], it is often regarded as an energy-onset response. However, there is evidence that the deflection is also sensitive to the other stimulus features, such as spectral composition [31], pure tone frequency [32, 33], fundamental frequency of harmonic tones [34] and temporal pitch extraction [27, 35, 36]. Furthermore, amplitudes of the N100m increase with pitch salience [27, 37] and fMRI studies show a correlate of the pitch salience with BOLD-responses in the non-primary auditory cortex [38]. Models with multiple dipoles have been succesfully used to separate specific energy and pitch responses [39].

In the present study we propose that N100m morphology reflects the processing of temporal asymmetry in auditory cortex. We hypothesise that amplitude differences observed in the transient response can be explained using adaptive, stimulus-dependent, windows of integration. Towards this goal, we first demonstrate the correlation between N100m amplitude and the perceived asymmetry in the salience of ramped and damped sinusoids. Second, we show how pitch perception models using adaptive integration windows can account for the processing mechanisms underlying the N100m deflection during temporal asymmetry perception. Our results suggest that the auditory system is capable to discern those two different sounds by continuously adapting the integration window of perceptual integration. Moreover, data also shows that temporal asymmetry is much more strongly represented in the right hemisphere than in the left hemisphere.

## Materials and Methods

### Experimentation

The study and all the measurements were approved by the ethics committee of the Heidelberg University’s Medical School and conducted with written informed consent of each subject.

#### Subjects

13 subjects were included in the perceptual study (aged between 24 and 37 years old) and 27 subjects participated in the neurophysiological experiment (aged 22-44 years old). All subjects reported normal hearing and had no history of audiological or neurological deficits. All of them were familiar with MEG recordings and psychoacoustic procedures. Measurements were approved by the local ethics committee and conducted with informed consent of each subject.

#### Stimuli

Experimental stimuli were ramped and damped sinusoids (see Fig 1) generated according to the parameter specifications described in [1] using a 1000 Hz carrier and an exponential amplitude envelope given by:

$$\begin{array}{c} E\left(t\right)=\sqrt{\frac{1}{T_{1/2}}}\;e^{-\frac{t\;\log2}{T_{1/2}}} \end{array}$$ (1)

Fig 1 illustrates two cycles of the modulated sinusoids. Stimuli consisted of a total concatenation of 20 cycles, as has been commonly done in human psychophysics and animal recordings using ramped and damped sinusoids [1, 23]. The length of one cycle was set to 50 ms to ensure that the discontinuity in the envelope at the end of each modulation cycle occurs at an upward-going zero-crossing of the carrier, so all stimuli present the same onset phase. Therefore, stimuli duration added up to a total of 1 s.

Half-life times (*T*_1/2_) of the modulator were 0.5 ms, 1 ms, 4 ms, 16 ms and 32 ms, respectively. To obtain approximately constant loudness for all conditions and minimise undesirable artefacts on the neuromagnetic signal, the amplitude was normalised by a factor proportional to the square root of the stimulus half life time [1].

#### Perceptual measurements

Psychoacoustic measurements of the paired comparison task were carried out using the temporally asymmetric sounds described above. Sounds were delivered through K240-DF headphones (AKG Acoustics, Vienna, Austria) at a level of 65 dB (SPL). Stimuli were presented in a single block of trials for each part of the experiment and listener. In each block, all possible combinations of pairs of non-identical stimuli (45) were presented in both orders. Thus, the psychoacoustic test consisted of 90 trials per block. For each trial, listeners had to indicate in a two-alternative task without feedback which sound of the pair was more tonal. After a training session, blocks were run just once. A scale for the relative pitch salience was derived from the results of the paired comparison experiment, using the Bradley-Terry-Luce (BTL) method [40]. This method allows to order the carrier salience of the temporally asymmetric stimuli on a perceptual scale. To analyse the results, we used the temporal asymmetry index defined in Eq (2) with *x* = *S* (*AI*_*S*_), where *S* denotes the relative pitch salience measured with the BTL method.

#### Neuromagnetic data recording and processing

Stimuli were presented diotically at an intensity level of 65 dB SPL using ER-3 transducers (Etymotic Research, Inc., Elk Grove Village, IL) connected to 90 cm plastic tubes and foam ear pieces. The sampling rate was set to 48 kHz. The order of the stimuli was randomized. ISIs were randomised between 1.0–1.1 s. The MEG session consisted of 120 trials for each condition.

Gradients of the magnetic field were acquired with a Neuromag 122 whole-head MEG system (Elekta Neuromag Oy, Helsinki, Finland) inside of a magnetically shielded room (IMEDCO, Hägendorf, Switzerland). Subjects sat in an upright position and watched a silent film of their own choice. Since neural mechanisms underlying pitch processing seem to evoke equivalent fields on attentive and inattentive subjects [27, 41], we chose to separate the psychophysical task from the MEG recordings in order to maximise the number of trials per session. Note that animal recordings on the same stimuli (e.g. [23]) were performed under anaesthesia and obviously also without a task.

The sampling rate was 1000 Hz and a bandwidth ranging from 0.01 Hz to 330 Hz. Auditory evoked fields were averaged over an epoch of from -500 ms to 1400 ms. Off-line averaging with artefact monitoring was performed using BESA 5.1 software (BESA Software, Gräfelfing, Germany). Epochs containing signals exceeding an absolute level of 8000 fT/cm and a gradient of 800 fT/cm per sample were discarded automatically, resulting in about 5% rejection rate. The baseline was calculated over the 100 ms interval prior to tone onset.

T1-weighted magnetic resonance images (MRI) were obtained from 10 of the listeners on a Magnetom Symphony 1.5 Tesla scanner (Siemens, Erlangen, Germany). Scans were performed in 176 sagittal slices yielding an isotropic voxel size of 1 mm^3^. Three-dimensional reconstructions were computed using the BrainVoyager software (version 4.4, Brain Innovation, Maastricht, The Netherlands). Dipole positions for these subjects were co-registered onto the individual MRI and then transformed into the standard space of Talairach [42] to illustrate the location of the generators (see, for instance, [43]). Since MRI images were not available for the remaining subjects, the spherical model was used without co-registration for 17 of the listeners. This method typically yields accurate locations for the N100m dipoles.

#### Neuromagnetic data analysis

In order to setup a model for the N100m we applied a spatio-temporal model [41] with one equivalent dipole per hemisphere. Dipole fits were based on the pooled 16 ms and 32 ms ramped and damped conditions since these sounds elicited a clear tonal percept. Fits were performed using unfiltered data and the fitting interval was about 30 ms around the peak of the N100m for each subject. A symmetry constraint was applied in 8 of 27 subjects. No further constraints concerning orientation or location of the equivalent dipoles were used. This method provided stable models for all subjects and was used as a spatio-temporal filter to derive the source waveforms of all 10 conditions. A principal component was computed over the last 100 ms of the epoch for each condition in order to compensate for drift artefacts [44]. This procedure, applied to each subject, assumed that the N100m response is evoked by the same generators in the auditory cortex; i.e. that the location and orientation of the equivalent dipole remain constant. This assumption is reasonable for this family of stimuli, since they all evoked the same pitch value.

Identical procedures were followed to compute an equivalent dipole model for the sustained field (SF), but the asymmetry constraint was applied only in 1 of the 27 subjects. The interval used to fit the SF dipoles covered the DC portion of the field, spanned from 500 ms to 1000 ms after tone’s onset.

In order to quantify ramped/damped asymmetry we used the temporal asymmetry index (*AI*_*x*_) [14]:

$$\begin{array}{c} AI_{x}=\frac{\left(x_{r}-x_{d}\right)}{\left(x_{r}+x_{d}\right)} \end{array}$$ (2)

where *x*_*r*_ and *x*_*d*_ denote the magnitude associated with ramped and damped stimuli respectively. To quantify the asymmetric behaviour in the amplitude of the N100m, we used Eq (2) with *x* = *M*, the amplitude of such component in the measured evoked fields.

Individual source waveform estimates were used to assess the N100m difference between conditions. Peak amplitudes were assessed using such averaged waveforms. Critical *t*-intervals were computed using the resulting distribution of the minima and the surrounding points in a 15 ms interval for each subject. A similar procedure was used to assess the properties of the sustained field, pooling the data points on the interval spanned between 800 ms and 1000 ms after tone’s onset.

### Modelling

We attempted to model the relationship between the dynamics of the N100m and perception by using two complementary models of pitch perception employing stimulus-dependent integration windows. Software for both models is freely available in http://www.pdn.cam.ac.uk/groups/cnbh/research/aim.php for the AIM and in http://sourceforge.net/projects/topdownpitchmodel/ for the GPM.

#### Auditory Image Model

**Fig 2:**
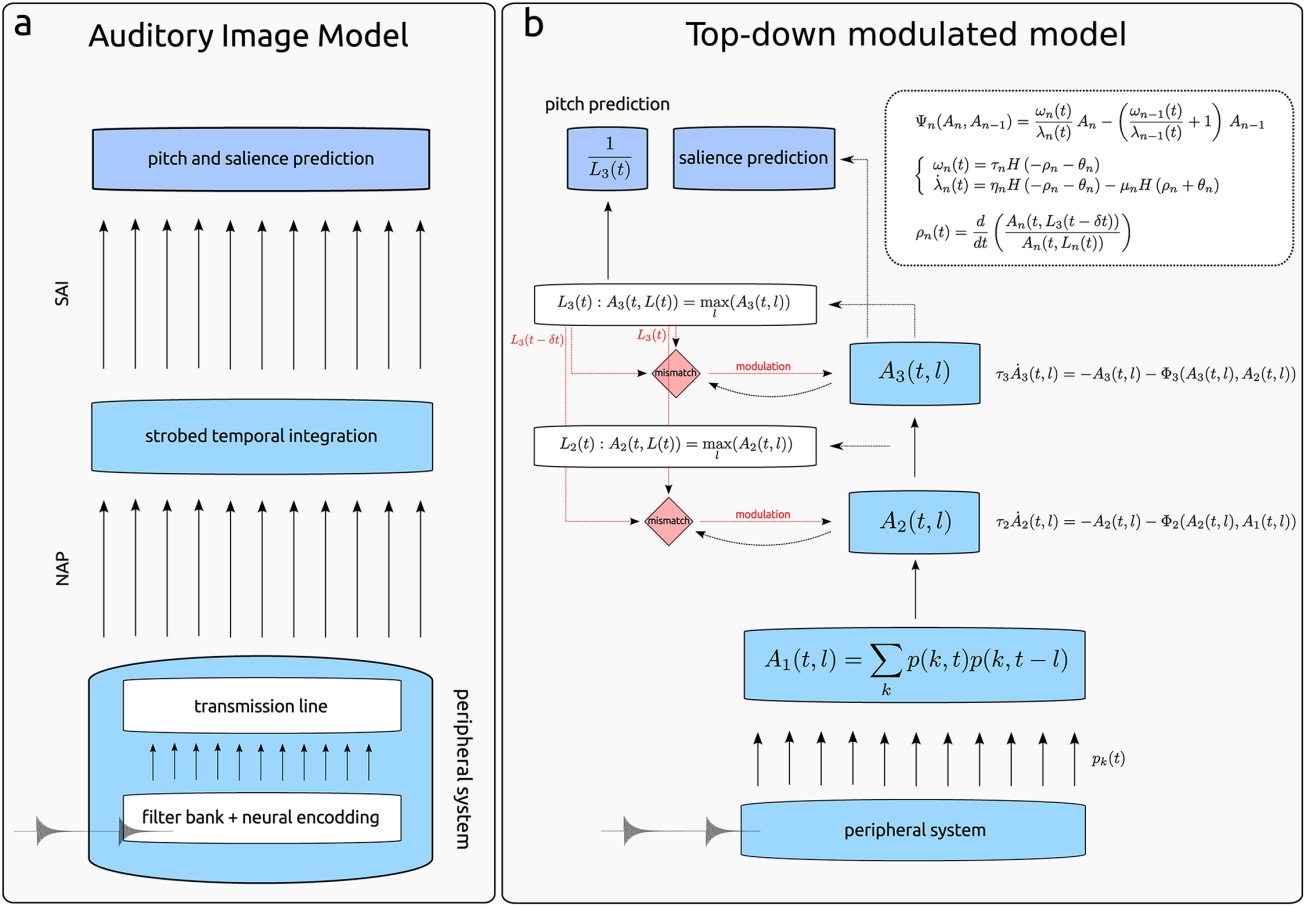
Schematic diagram of the Auditory Image Model and the Top-down Modulated Hierarchical Model of Pitch. a) Schematic view of the Auditory Image Model (AIM) [14]. In the first stage, peripheral auditory filters transform the input waveform into a multi-channel representation of basilar membrane motion. The next stage applies a hair cell model and converts this motion into a neural activity pattern in the auditory nerve (NAP). In the final stage, this signal is used to produce a stabilised representation of the stimuli by means of strobed temporal integration. The output of this process is termed the stabilised auditory image (SAI) of the input stimulus. b) Schematic view of the top-down modulated Hierarchical Generative Model of pitch perception (GPM) [17]. The peripheral processing is similar to the one in AIM (bottom). The next step consists of a coincidence detection process of auditory nerve activity patterns for different cochlear delay lines *l*, *A*_1_(*t*, *l*). Further processing is carried out by two consecutive ensemble models *A*_2_ and *A*_3_ performing leaky integrations of input activity using time-varying integration windows. Such ensembles correspond putatively to pre-thalamic and central auditory areas. A top-down, stimulus-dependent mechanism modulates the size of the effective integration windows of bottom-up information.

The Auditory Image Model (AIM) [14] consists of three sequential transforms associated with three different processing stages of the ascending auditory pathway, two at the peripheral auditory system and one at a central stage as illustrated in Fig 2a. AIM was originally designed to simulate a highly idealized neural representation of auditory stimuli, assumed to underlie the first conscious awareness of sounds [14].

**Fig 3:**
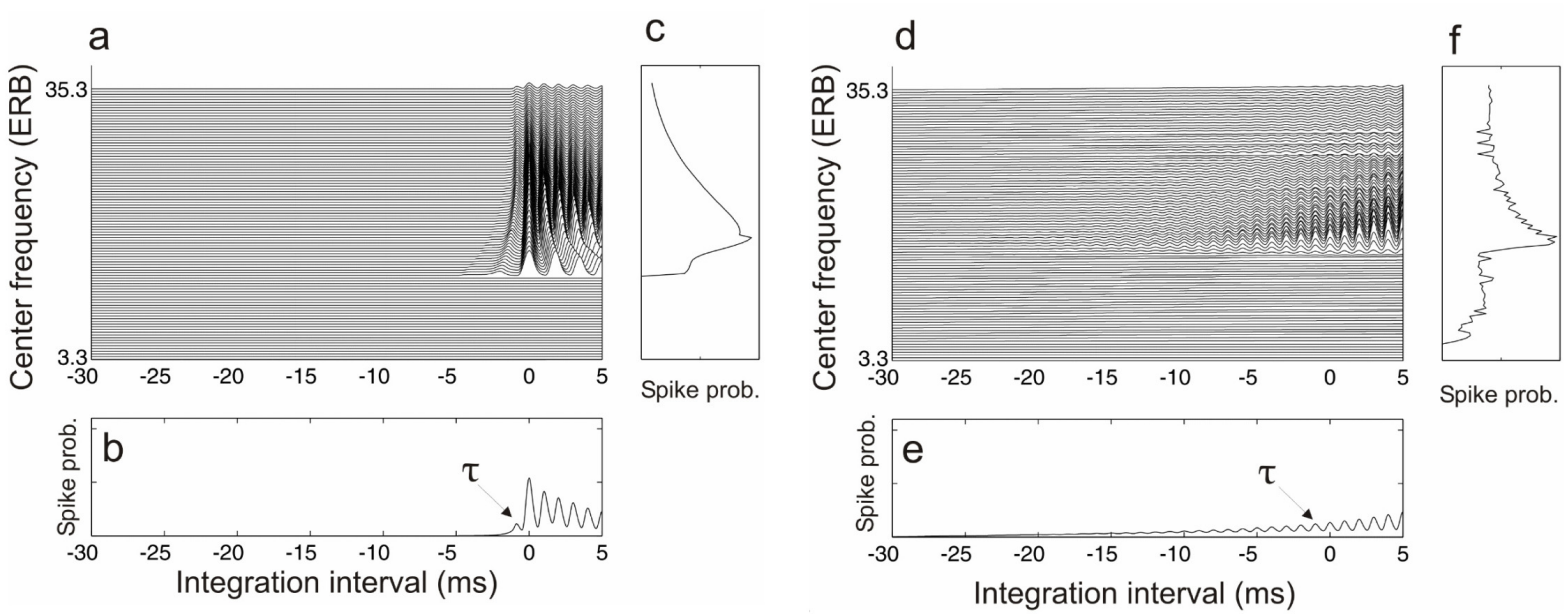
Output of the auditory image model for the *T*_1/2_ = 4ms ramped and damped sinusoids. Auditory Image Models’ output for damped (a–c) and ramped (d–f) trains (*T*_1/2_ = 4ms) at the time point of the same envelope height. Panels a) and d) show the stabilized auditory image (SAI) over time in each cochlear frequency channel. Panels c) and f) represent the spike probability averaged over time. Panels b) and e) show the summarised activity of all channels in the auditory image. The integration interval is the inverse of the carrier frequency applied [11], thus it shows a peak at *τ* = −1ms in the figure. The height of this peak predicts the perceived carrier salience.

The first stage of AIM uses a non-linear transmission-line filter bank, accounting for the spectral analysis performed in the cochlea in the range of 100–10000 Hz [45]. The simulations in the present study were carried out using 100 channels. During the second stage, the basilar membrane motion is converted into a multi-channel pattern which simulates the neural activity (NAP) of the auditory nerve (shown for ramped and damped sounds in Fig 3a). In the third stage of AIM, the spike probability *p*(*t*, *k*) for each channel *k* at each time point *t* is transformed into an interpretable representation: the *stabilised auditory image* (SAI).

This last transformation is carried out by means of a mechanism called *strobed temporal integration*, operating independently in each cochlear channel by transforming a spike-train signal into a time-interval signal. When a pulse is detected in the spike train of each channel (i.e. when the value of the signal exceeds some adaptive threshold which is asymmetric in time, see Fig 3b) the signal is copied point by point into the buffer. This mapping continues until a new pulse exceeds the adapted threshold. Then a new strobe is triggered and the signal is transferred to the buffer. The buffer decays within 30 ms. This decay allows the system to respond to rapid stimulus changes. The integrated SAI provides complete information about the perceived pitch and its strength. Time-interval repetitions along time are represented in the first peak in the SAI, whose position in the time-interval space represents the perceived pitch of the stimulus [46]. Similarly, the ridge height is related to its pitch strength [47]. Therefore, we can use the mean value across cochlear channels of the height of the first peak of the SAI to extract a prediction of the perceptual pitch salience from the model. Note that, as a consequence of the peripheral preprocessing and the adaptive strobed integration, the SAI is not a simply spectral decomposition of the waveform of the stimuli, but the result of an elaborated nonlinear transformation that reflects the pitch elicited by the stimuli [46].

A key feature of the peak detection during the strobing process is that the threshold is adaptive, such that the rising envelopes of ramped sounds provide multiple snapshots of activity in a channel whereas decaying envelopes as given by damped sounds exhibit just a relative small number of strobes. This effect is illustrated in Fig 3b and 3c, which show the different strobing for a sinusoid with *T*_1/2_ = 4ms and the resulting SAI with a much larger peak height for the ramped sound.

#### Top-down modulated model

A hierarchical model of interacting neural ensembles incorporating a top-down modulation process (top-down modulated model of pitch perception, or *generative* pitch model in short, GPM [17, 48]) was used for further analysing the role of adaptive integration windows in the perception of ramped and damped sinusoids.

Similarly to AIM [14], and to the so-called autocorrelation models of pitch [11, 13], the top-down model receives its input from the hair cell transduction model [49], which generates the auditory-nerve spike probabilities *p*(*t*, *k*) as a function of time *t* in each cochlear frequency channel *k*. The GPM consists of a cascade of three layers of activation with time-dependent outputs *A*_1_, *A*_2_ and *A*_3_. The output of the first stage represents the probability of generating two spikes delayed by a certain lag *l* across all channels:

$$\begin{array}{c} A_{1}\left(t,l\right)=\sum_{k}p(t,k)p(t-l,k) \end{array}$$ (3)

The sum of this quantity for the stimulus onset *t* = 0 to *t* weighted by an exponential decay function renders the summarized autocorrelation function (SACF) [11]. The value for the lag *l* where SACF = ∑_*t*_ *A*_1_(*t*, *l*) reaches its maximum represents the pitch value in autocorrelation models [11, 13], whilst the pitch strength is often represented in the difference between SACF(*t*, *l*_max_) and the value of SACF(*t*, *l*) at the second highest lag. However, these models fail to explain a large range of pitch phenomena [17] requiring a more realistic processing. In this model, this is solved using a leaky integration process implemented in the superior two layers, endowing a top-down mechanism in order to control the size of the integration windows (see Fig 2b). The integrators are implemented as a cascade of two highly idealised neural ensemble models [17, 50] with top-down recurrent connections modulating the size of the integration windows.

The activity at the second processing stage *A*_2_(*t*, *l*) (see Fig 2b) is computed as a nonlinear leaky integrator of the activity at the previous stage *A*_1_(*t*, *l*), using a lag-dependent short time constant 2ms ≤ *τ*_2_ ≤ 100ms [51]. This activity represents the firing rate of a set of auditory nerve fibres receiving inputs from different delays *l*. Overall, the output of this stage simply represents a periodicity extraction averaged along channels using a short exponential decay, like the one used in [11]. This stage mirrors processing carried out by sub-thalamic neural populations [49, 52, 53].

The subsequent, last third stage *A*_3_(*t*, *l*) implements a low-pass filter of short-term periodicities encoded in *A*_2_(*t*, *l*) using a long time scale *τ*_3_ (typically, *τ*_3_ ≥ 100ms) and a nonlinear activation function which is briefly discussed in the next section. This processing is assumed to be located more centrally in the brain. This kind of hierarchical architecture embodying multiple time scales is fully in line with observations of functional magnetic resonance imaging studies (e.g. [54, 55]).

Both integration stages are implemented as simple time-varying exponential averages:

$$\begin{array}{c} A_{n}\left(t,l\right)=A_{n}\left(t-\Delta t,l\right)e^{-\frac{\Delta t}{E_{n}\left(t\right)}}+\frac{1}{g_{n}\left(t\right)}\frac{\Delta t}{\tau_{n}}A_{n-1}\left(t,l\right)\Delta t\;n=2,3 \end{array}$$ (4)

where Δ*t* is the time step of the integration, *g*_*n*_(*t*) is a normalisation factor and *E*_*n*_(*t*) is the effective integration window of the *n*th stage, represented as the instantaneous exponential decay rate of the response at the *n*th integration stage (*E*_*n*_(*t*) ≤ *τ*_*n*_ for *n* = 2,3 and *E*_1_ ≡ *τ*_1_ = 1).

Similarly to AIM, the lag in which the output at the final processing stage *A*_3_(*t*, *l*) is maximum will be denoted as *L*_*n*_(*t*) throughout the work, and will be referred to as the lag prediction. Therefore, 1/*L*_3_(*t*) represents the predicted pitch at time *t*. Equivalently, we define the expected pitch as the pitch prediction at the previous time step 1/*L*(*t* − Δ*t*) [17].

Crucially, the effective integration *E*_*n*_(*t*) windows are not static. Instead they are adaptive and top-down driven, which permits to detect unexpected changes in the input stimulus (such as the offset of a tone in a sequence). In AIM (see Methods), information about past events is integrated until the auditory image is *stable*, and then the adaptation is performed with an exponential decay across time. Consistently, in the GPM model, the integration windows decay rapidly during periods where either there is a sudden discrepancy between the pitch prediction 1/*L*_*n*_(*t*) and expectation 1/*L*_*n*_(*t* − Δ*t*) or there is a long sustained period with no discrepancies between them (see next section and [17] for further details).

#### Parallels with neural ensemble models

The idealized GPM can also be understood in terms of neural ensemble models. Taking the limit Δ*t* → *dt*, the modulated cascade of integrators is equivalent to a hierarchy of neural ensemble models of the Wilson-Cowan type [56]:

$$\begin{array}{c} \tau_{n}{\dot{A}}_{n}\left(t,l\right)=-A_{n-1}\left(t,l\right)-\Psi_{n}\left(A_{n}\left(t,l\right),A_{n-1}\left(t,l\right)\right) \end{array}$$ (5)

with the following activation function:

$$\begin{array}{c} \Psi_{n}\left(A_{n}\left(t,l\right),A_{n-1}\left(t,l\right)\right)=\frac{\omega_{n}\left(t\right)}{\lambda_{n}\left(t\right)}\;A_{n}\left(t,l\right)-\left(1+\frac{\omega_{n-1}\left(t\right)}{\lambda_{n-1}\left(t\right)}\right)\;A_{n-1}\left(t,l\right) \end{array}$$ (6)

The gains $\frac{\omega_{n}\left(t\right)}{\lambda_{n}\left(t\right)}$ in the activation function are modulated by the top-down mechanism, and at the same time modulate the effective integration windows in Eq (4):

$$\begin{array}{c} E_{n}\left(t\right)=\frac{\tau_{n}}{1+\frac{\omega_{n}\left(t\right)}{\lambda_{n}\left(t\right)}} \end{array}$$ (7)

In the absence or deactivation of the top-down mechanism, $\frac{\omega_{n}\left(t\right)}{\lambda_{n}\left(t\right)}=0$ and the integration windows are set at a fixed time. The top-down mechanism gets activated when a mismatch between the expectation and prediction of pitch occurs, by setting the gains to a positive value and thus decreasing the size of the integration windows. Full details of the mechanism can be found in the original publication [17].

In summary, the shape of this model preserved certain constraints established in neural ensemble theory. This model has been shown capable of explaining a wide range of pitch perception phenomena, including the balance between temporal integration and resolution of pitch perception [17]. Thus, it is worth investigating whether it can predict effects of temporal asymmetry like AIM [14].

#### Top-Down modulation and the N100m

The GPM approach has been shown to be consistent with available neuroimaging data associated to the perception of Iterated Ripple Noise pitch [27]. More precisely, the derivative of the model output at the predicted pitch *L*_3_(*t*), *A*_3_(*t*, *L*_3_), was closely correlated with the latency of the N100m component of the evoked responses in antero-lateral Heschl’s Gyrus (see [17, 27] for details).

Hence, in the present study, we evaluated the capacity of this model for further explaining electrophysiological results by comparing the dynamics of the top layer neural ensemble, representing activity in auditory cortex, with the morphology of the N100m response evoked by the each of the ramped and damped stimuli. The analysis was performed for the 10 stimuli considered in the experimentation (five different *T*_1/2_ for each, ramped and damped envelope; see Fig 1). For each of the sounds, we matched the response of the model’s top layer at the pitch value prediction *A*_3_(*t*, *L*_3_(*t*)) to the amplitude of the evoked response within a time window of 50 ms surrounding the N100m peak. To fit the peak, we proposed a linear relationship between the amplitude of the model and the amplitude of the MEG signal (see e.g. [57]).

*N*-fold cross validation was used to robustly compute the parameters of the transformation: we performed an individual linear fitting for each of the *N* = 27 subjects in the experimentation. Then, parameters of the linear fits were fixed and tested using the evoked fields of the remaining *N* − 1 subjects, yielding to a total of *N*(*N* − 1) = 702 cross-validation folds per stimuli. This procedure enabled a highly robust statistical assessment.

### Statistical testing

Correlations shown in the Results section were computed using the Pearson’s coefficient. *p*-values were obtained using non-parametric Wilcoxon rank-sum tests, since samples were generally non-Gaussian distributed (normality was assessed according to *χ*^2^ and nonparametric Lilliefords tests, and accepted at *p* < 0.001).

## Results

### Experimental results

#### Psychoacoustics and evoked responses

**Fig 4:**
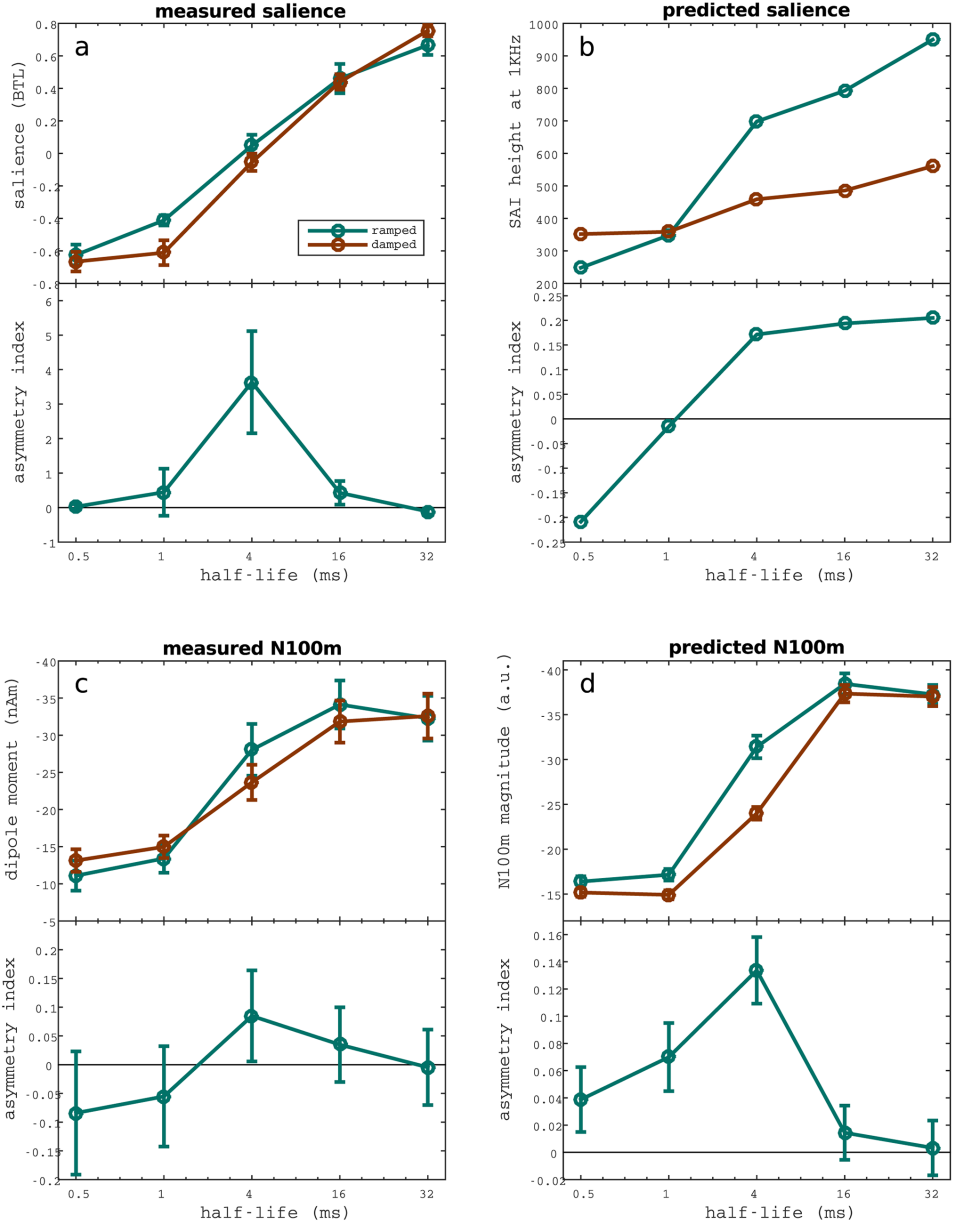
Comparison of the perceived salience, N100m magnitude, and the prediction of the two models of pitch. Perceptual and neuromagnetic results for each of the five pairs of ramp and damp stimuli. The corresponding temporal asymmetry indices are drawn at the bottom of each plot (see Eq (2)). (a) Perceived salience estimated by the BTL method and averaged across subjects (*N* = 13). (b) SAI mean ridge height at the frequency of the carrier (1 kHz). Ridge height was used to predict the perceived salience of the stimuli [14]. (c) Magnitude of the N100 component averaged across subjects. (d) Top-down modulated model’s predictions for the amplitude of the N100m peak, computed as a linear transform of the derivative of the activation of the top layer population evaluated at the winning frequency. The linear relationship was cross-validated across subjects (see Methods), yielding to a total of 702 predictions. The figure shows the average of the predictions. Significant correlations were found between perceived saliency 4a) and N100m magnitude (4c); between the perceptual observations 4a AIM responses (4b) and between the N100m magnitude 4c) and GPM predictions (4d). Error bars represent SME.

Fig 4a shows perceptual responses as a function of the stimulus’ envelope’s *T*_1/2_. Pitch salience increased with *T*_1/2_ values for both, ramped and damped sounds, but the pitch of the ramped tones was generally judged as more salient than the pitch of their damped counterparts. This difference reached significance for the critical value *T*_1/2_ = 4ms (*p* = 0.0077, *n* = 13) and for *T*_1/2_ = 1ms (*p* < 0.001, *n* = 16). The difference is attenuated and remains not significant for the rest of the conditions. This behaviour is also reflected in the salience asymmetry index *AI*_*P*_ (see Fig 4a). Note that the behaviour of the temporal asymmetry index is not well defined over values near zero, which accentuates the difference between ramped and damped at 4 ms. For that reason, statistical significance was not measured using the temporal asymmetry index but rather using the raw BTL perceptual data.

#### Neuromagnetic data

In the next step we analysed the neuromagnetic data in order to find a correspondence between the perceived salience and the morphology of the N100m deflection. Approximate Talairach-coordinates [42] for the sources of the N100m as given by the standard BESA spherical model were localised in lateral Heschl’s gyrus (left: *x* = −48 ± 1, *y* = −24 ± 2, *z* = 0 ± 2; right: *x* = 49 ± 1, *y* = −23 ± 2, *z* = 0 ± 2).

**Fig 5:**
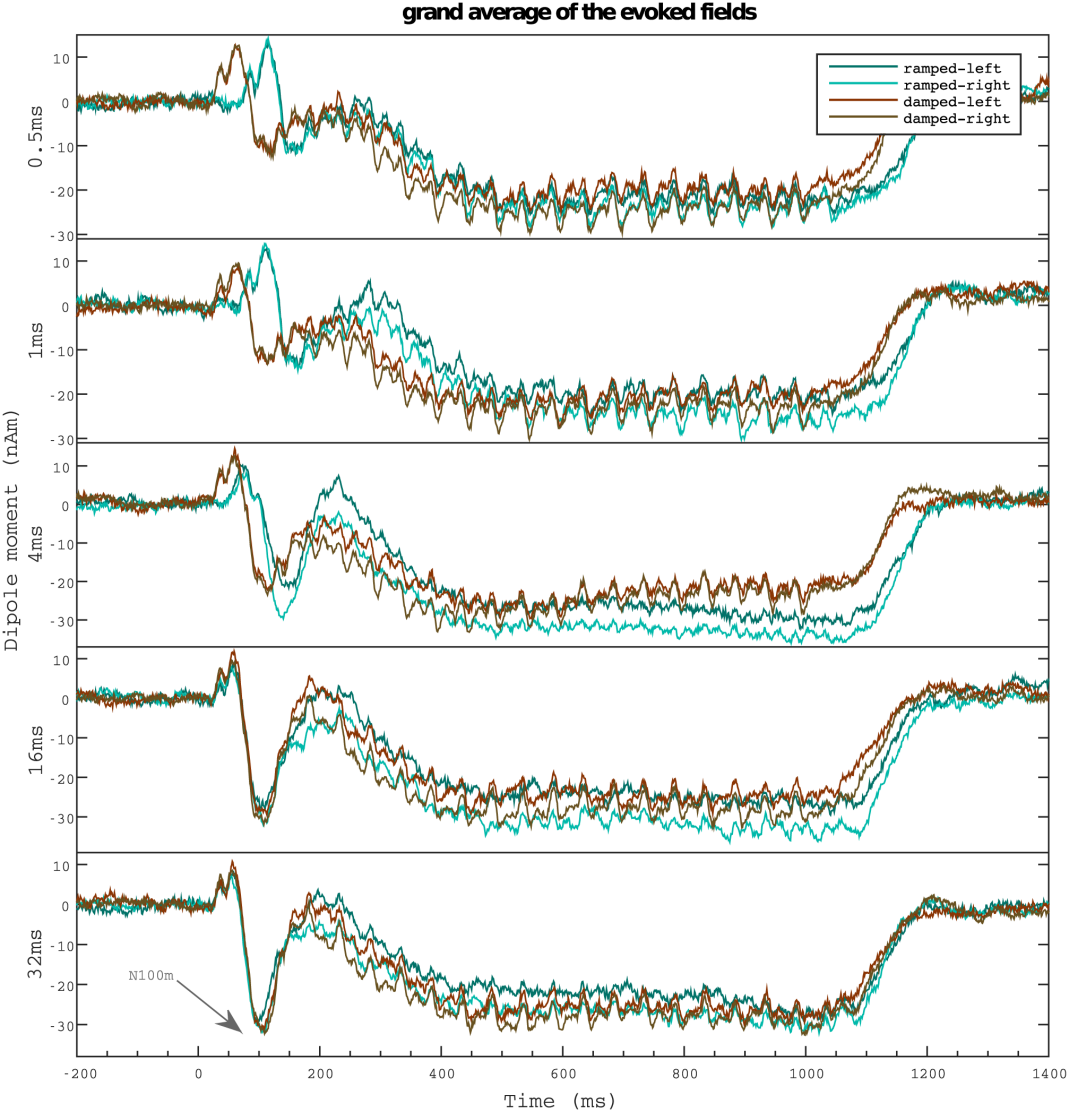
Auditory fields evoked by the ramped and damped sinusoids. Grand mean source waveforms for the five different conditions of ramped and damped sinusoids. Average was taken over subjects (*n* = 27) for both hemispheres. The magnitude of the N100m increases for rising *T*_1/2_ values of the stimuli. Note the maximal difference between ramped and damped sinusoids in the right hemisphere for the *T*_1/2_ = 4ms condition.

The cortical responses are summarised in Fig 5. Source waveforms showed a prominent N100m followed by a large sustained field. Due to the envelope structure of the ramped sounds, latencies of the corresponding responses were delayed in comparison to their damped counterparts.

N100m amplitudes were assessed using the averaged responses across hemispheres. As shown in Fig 4c, the peak amplitude increased with the *T*_1/2_ of the stimuli for all conditions and was significant for the transition from *T*_1/2_ = 1ms to higher half-life values (ramped: *p* = 0.0003, *n* = 837; damped: *p* = 0.0039, *n* = 837) and for the transition from *T*_1/2_ = 4ms to higher half life times in the damped case (*p* = 0.0146, *n* = 837). Consistently with perceptual results, ramped tones evoked larger N100m than damped ones, with a maximal difference at the critical value of *T*_1/2_ = 4ms (*p* = 0.0008, *n* = 837). Accordingly, the corresponding temporal asymmetry indices *AI*_*M*_ (also shown in Fig 4c), exhibited a maximum for *T*_1/2_ = 4ms, fully in line with the perceptual results shown in Fig 4a.

According the the standard BESA spherical model, sources of the sustained field were located more medially but near the N100m sources, in fully agreement with previous studies in pitch [39] (left: *x* = −45 ± 1, *y* = −23 ± 2, *z* = 3 ± 2; right: *x* = 45 ± 1, *y* = −20 ± 2, *z* = 2 ± 2). Waveform morphologies were similar to the fields observed in the N100m model in all the conditions and thus they are not shown in a separate plot.

Sustained fields’ behaviour mimicked the trends of the N100m. Significant correlations were found between SF’s average depth and N100m amplitude (ramped: *R* = 0.9247, *p* = 0.0245; damped: *R* = 0.9744, *p* = 0.049). Correspondingly, damped responses were shallower than ramped responses for the five half-life times (*p* < 0.0001, *n* = 5427). SF’s depth also increased with the *T*_1/2_ of the stimuli for all conditions, and it was significant for the transition from *T*_1/2_ = 0.5ms to higher half-life values (ramped: *p* = 0.0244, *n* = 5427; damped: *p* < 0.0001, *n* = 5427); for the transition from *T*_1/2_ = 1ms to higher half life times (ramped and damped: *p* < 0.0001, *n* = 837); and for the transition from *T*_1/2_ = 4ms to higher half life times in the damped case (*p* < 0.0001, *n* = 5427).

#### Correlation between neuromagnetic and perceptual responses

Taken together, these results show a high correlation between the magnitudes of N100m and the relative perceived carrier salience. This linear correlation was quantitatively measured using the Pearson’s correlation coefficient between the BTL salience scores and the magnitude of the N100m for ramped (*R* = −0.9597, *p* = 0.0097) and damped (*R* = −0.9867, *p* = 0.0018) stimuli.

#### Inter-hemispheric differences

**Fig 6:**
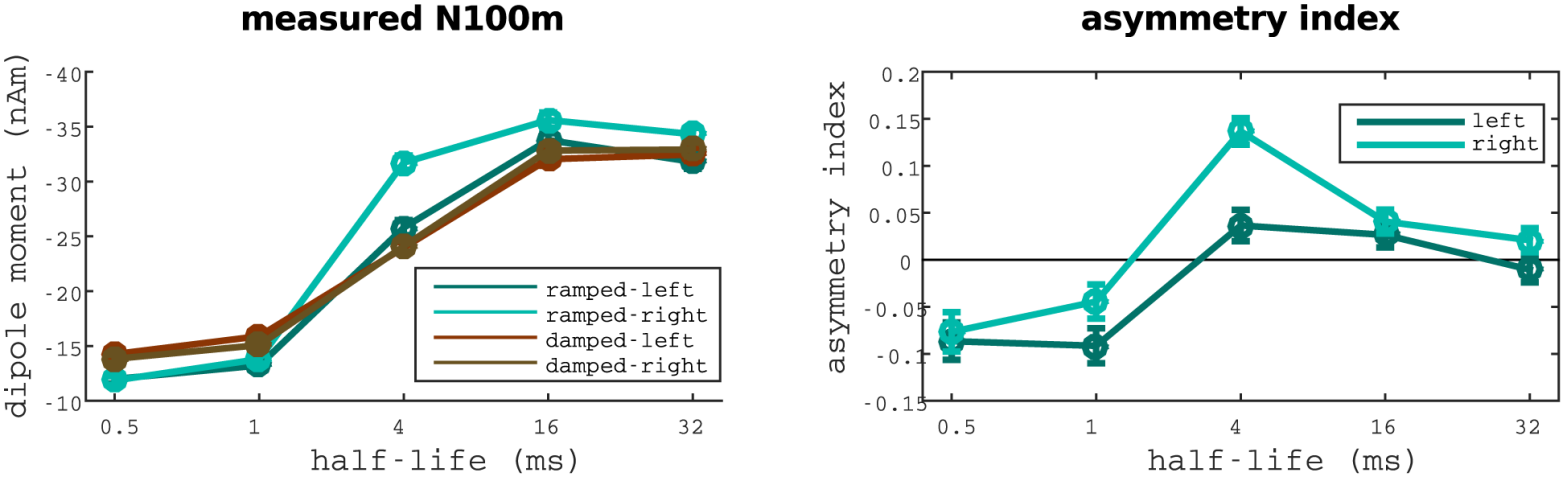
Inter-hemispherical differences observed between the fields evoked by ramped and damped sinusoids. Comparison between the fields evoked in left and right hemispheres for ramped and damped stimuli. N100m’s magnitude is plotted in the left panel. Corresponding asymmetry indices are displayed in the right panel.

Strong differences between hemispheres were observed in the evoked fields for the *T*_1/2_ = 4ms condition (see Fig 5). Specifically, the difference between ramped and damped sinusoids was much larger in the right than in the left hemisphere. To asses the size of the effect, we analysed the magnitude of N100m evoked in each hemisphere separately (see Fig 6). Strikingly, the difference between the N100m evoked by ramped and damped for the critical half-life of 4 ms was only significant in the right hemisphere (right: *p* < 0.0001, *n* = 837, left: *p* = 0.7124, *n* = 837); whilst differences between fields evoked by sinusoids modulated with different half-life values were similar in both hemispheres.

Correspondingly, we computed the difference between the N100m’s magnitude in left and right hemispheres for all the stimuli. The hemispheric asymmetry was, again, only significant for the *T*_1/2_ = 4ms ramped sinusoid (*p* < 0.0001, *n* = 837).

The sustained field showed similar hemispheric behaviour as the N100m amplitude. Correlations between these two magnitudes in each hemisphere were very high for ramped sinusoids (left: *R* = 0.9926, *p* = 0.0008; right: *R* = 0.9959, *p* = 0.0003), and smaller but still significant for the damped stimuli (left: *R* = 0.9251, *p* = 0.0243; right: *R* = 0.9322, *p* = 0.0210). Responses in the right hemisphere were generally larger than in the left hemisphere in all conditions (*p* < 0.0001, *n* = 5427).

### Model simulations

In this subsection we compare the simulation output of the models and compare these patterns with the psychoacoustic and neuromagnetic results.

#### Simulations with AIM and perception

The Auditory Image Model successfully accounted for the carrier salience for ramped and damped stimuli (see Fig 4b) as evidenced from the high correlation with the measured perceptual trends shown in Fig 4a (ramped: *R* = 0.978, *p* < 0.05; damped *R* = 0.978, *p* < 0.05). However, the temporal asymmetry index did not show a high amplitude with the perceptual results for large *T*_1/2_, predicting larger differences than observed in the experimentation. Still, AIM was able to predict the perception elicited by ramped and damped stimuli, suggesting that the strobed integration process effectively amplifies differences in responses at compared to the pattern at the level of the auditory nerve. The next question we will address is whether we can find a functional explanation to this effect in terms of top-down modulatory processes.

#### Top-down modulated model and neuromagnetic data

As a complementary analysis, the GPM model was used to predict the evoked response in the neighbourhood of the N100m deflection. Interestingly, this model provides a phenomenological explanation of the processing in central auditory stages in terms of top-down modulatory effects.

**Fig 7:**
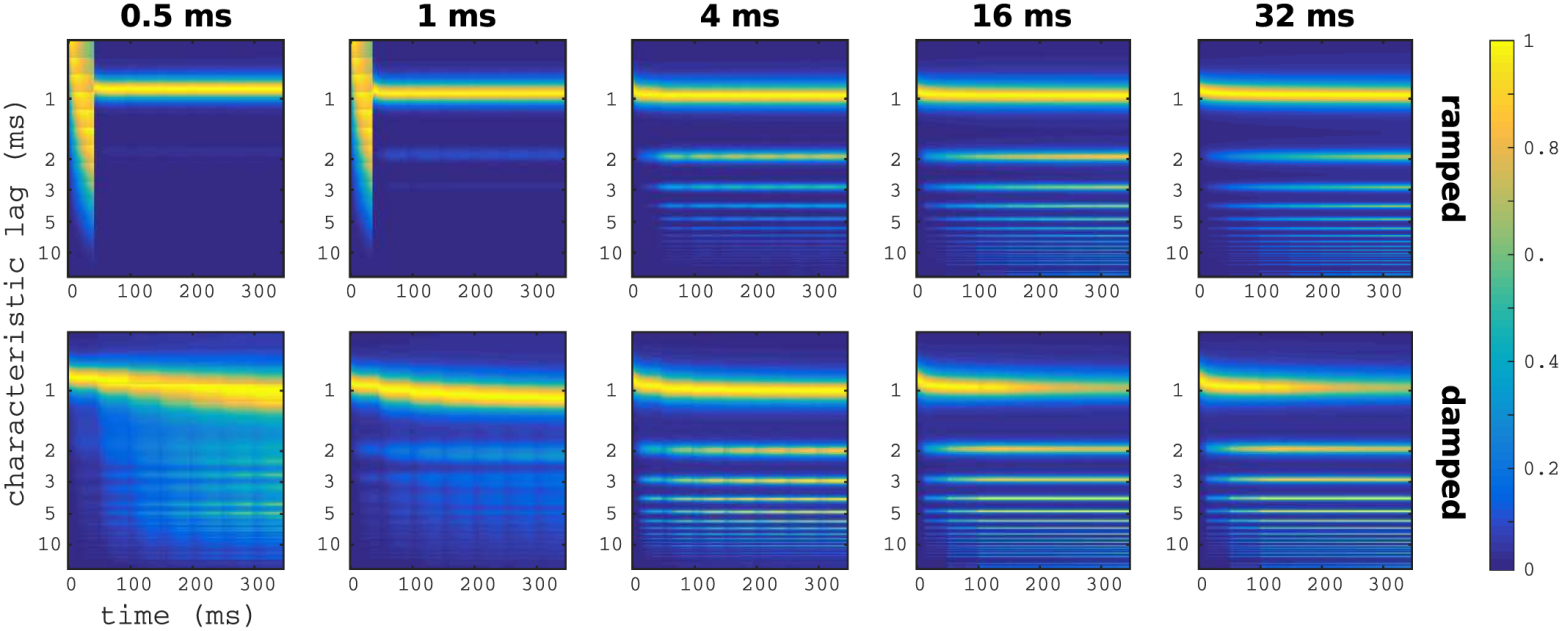
GPM raw output for the ramped and damped stimuli. Heat maps show the evolution in time (*x*-axis) of the activity of the different ensembles (*y*-axis) in the third layer of the GPM model for ramped (top) and damped (bottom) sinusoids with different *T*_1/2_. In all cases, after a small period of instability, there is a maximum centred in the ensemble characterized by *δt* = 1ms, the frequency of the carrier sinusoid. Qualitative differences are noticeable between the output of ramped and damped stimuli, and also between stimuli with different envelope time constants.

First, we computed the raw output of the model for the set of ramped and damped stimuli. As expected, and in agreement with AIM results, the model output shows a pronounced peak of activation in the 1 ms lag (corresponding to the frequency of the carrier). Moreover, perceptual differences are noticeable between ramped and damped stimuli, and between stimuli with different *T*_1/2_ (see Fig 7).

**Fig 8:**
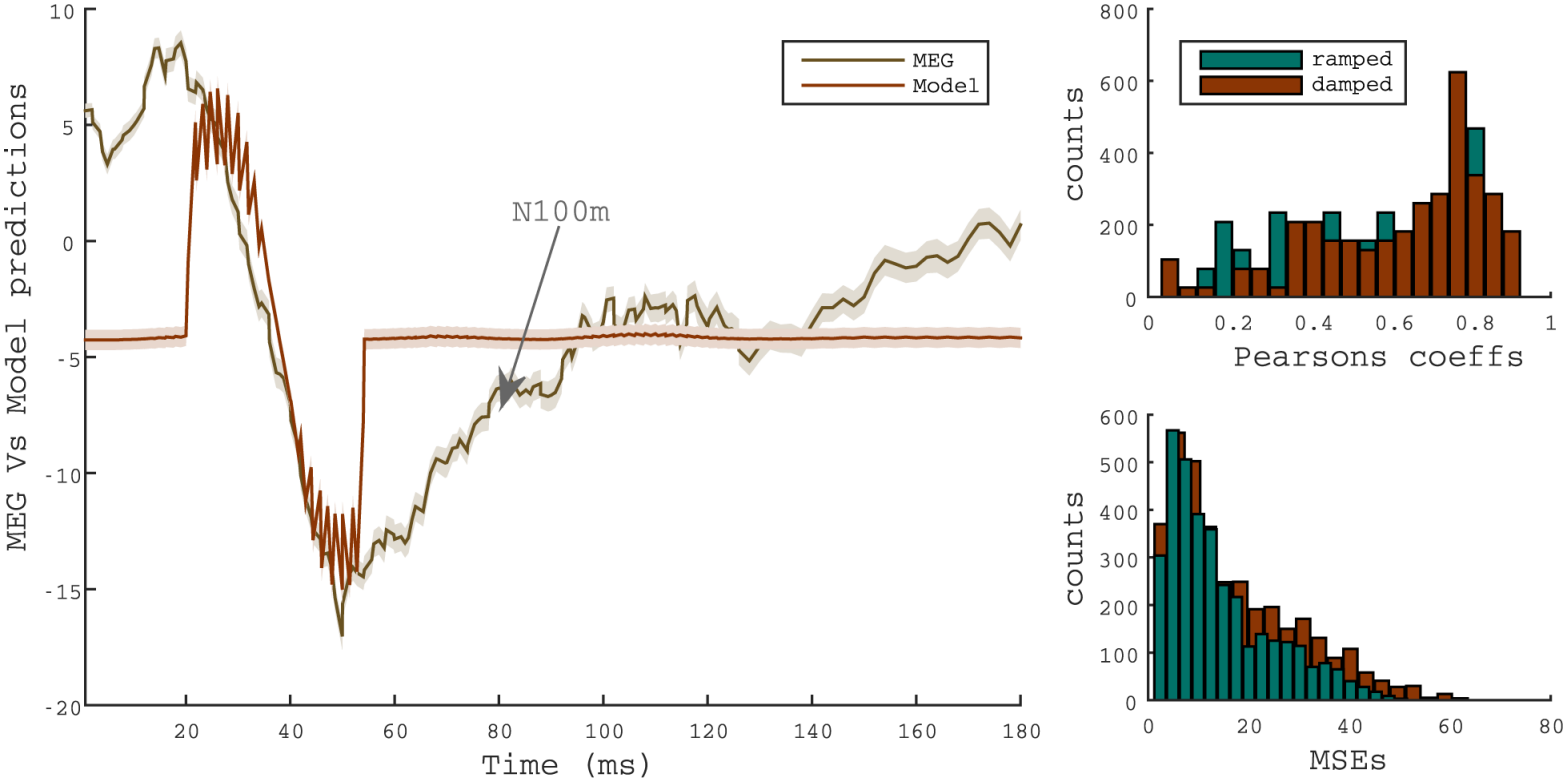
Summary of the statistics of the fit between the N100m transient and the output of GPM. Left panel: Example of the model response derivative, normalized to the amplitude of the recording, for a ramped stimulus (*T*_1/2_ = 0.5ms) and the corresponding recordings, averaged across right and left hemispheres and participants. Transparent shadows represent standard deviations. Right panel: Histograms of the Pearsons’s correlation coefficient and root-mean-square errors corresponding to the fittings between the GMP prediction and MEG recordings in an interval of 50 ms around the N100m peak. Each value corresponds to a single cross-validation instance for ramped and damped stimuli.

The GPM enables us to analyse correlations between the models’ ensemble dynamics and neuromagnetic data (see Methods). An example of such quantitative prediction is shown in Fig 8 for a ramped sound modulated by an envelope with *T*_1/2_ = 0.5ms. In the figure, the prediction is compared with the grand average of the auditory evoked fields. The simulation closely resembles the trend of the recorded activity, in particular with regards to the magnitude and latency of the N100m. More generally, the two histograms in Fig 8 show a summary of the model prediction fittings with MEG responses for all cross-validation combinations (see Methods). Although simulations do not show, in general, a close agreement with the observed overall waveform of the neuromagnetic recordings, small root mean square values were observed, with only a small bias error for short *T*_1/2_.

A systematic analysis of the N100m magnitude predictions for all stimuli is shown in Fig 4d. Consistently with perceptual results, differences between model simulations for ramped and damped stimuli are highly significant for a *T*_1/2_ = 4ms stimulus (*p* < 0.0001, *n* = 702). Moreover, results in Fig 4d show a strong linear correlation with the magnitude of the N100m observed in the auditory evoked fields (see Fig 4c) for both, ramped (*R* = 0.9972, *p* = 0.0002) and damped (*R* = 0.9899, *p* = 0.012) stimuli.

**Fig 9:**
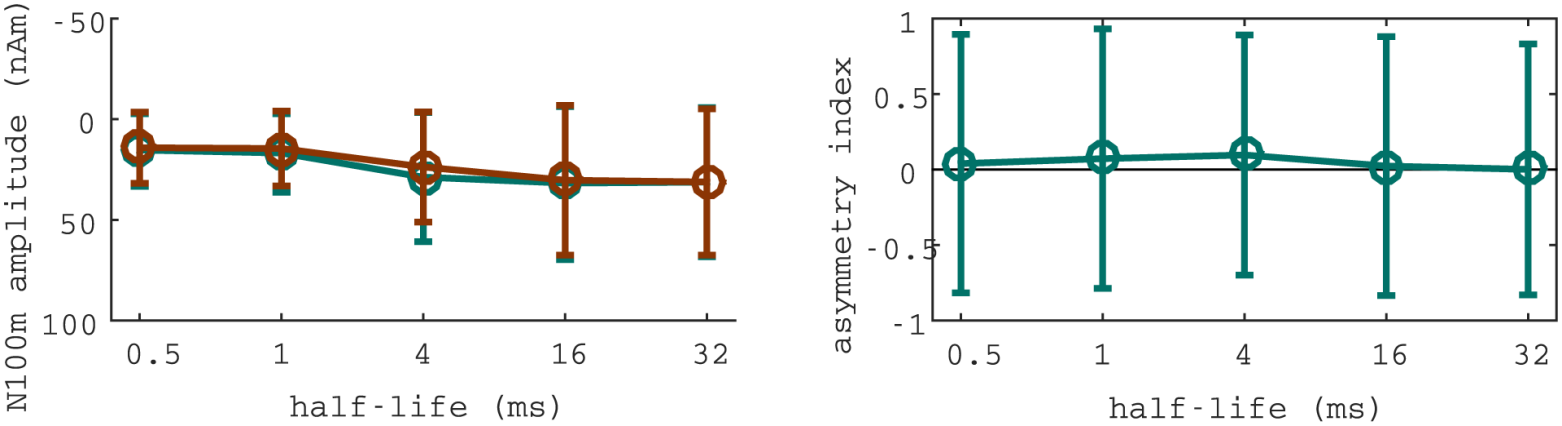
Autocorrelation model’s predictions for the amplitude of the N100m peak. Predictions were computed following the same procedure as in the analysis of the top-down modulated model (see Fig 4d). Predictions of the autocorrelation model do not show statistically significant correlations with the N100m values or the perceptual predictions. Moreover, the predicted amplitudes elicited by ramped and damped sinusoids with *T*_1/2_ = 4,ms are not significantly different in this analysis.

To test whether the adaptation of the temporal window of integration is necessary to successfully predict the N100m amplitude, we tried to replicate the previous results using an autocorrelation model without top-down modulation [13], effectively equivalent to the top-down modulated model introduced in Methods with static rather than adaptive integration windows *E*_*n*_(*t*). The analysis failed to produce significant results (see Fig 9), indicating that the top-down has a crucial role in the N100m dynamics elicited by this family of stimuli.

## Discussion

The aim of this study was to characterise the neuromagnetic representation of auditory temporal asymmetry in human auditory cortex and to compare these neurophysiological responses with perceptual data and computer simulations of perceived pitch. We found that the N100m magnitude was closely correlated with perceived pitch salience. Furthermore, N100m amplitudes were closely related to the computer simulations of the classical Auditory Image Model [14] as well as the hierarchical top-down modulated model of pitch (GPM) [17]. The latter enabled us to provide a phenomenological understanding of bottom-up and top-down processes which may underlie the neural coding of perceived temporal asymmetry.

The present study extends the work of Patterson and colleagues [14] by analysing the auditory evoked fields elicited by the same set of ramped and damped in human listeners. We observed that the amplitude of N100m increased with stimulus’ *T*_1/2_ for all conditions and both hemispheres, thus providing a neurophysiological correlate of the actual strength of the tonal component. The morphology of the N100m source waveforms strongly varied as a function of the temporal features of the envelope (see Fig 5), especially for the critical *T*_1/2_ = 4ms pair of stimuli, again in full agreement with subject’s perceived tonality.

It is important to note that subcomponents of the N100m exhibit different temporal integration times [39, 58]. However, the location of the N100m sources found in this work are located in alHG, and hence we can safely assume that we assessed pitch related generators, as reported in humans [27, 39] and animal studies (e.g. [59]).

It is also noticeable that we observed a tight correlation between the psychophysical data, which was based on judgements of 20 modulation cycles lasting 1000 ms overall, and the N100m, peaking at about 100 ms after sound onset. Presumably, the N100m reflect mechanisms affecting only the beginning of the sound, revealing that processes occurring at the onset of the stimuli are crucial for the decoding of temporal asymmetry.

The observed results are also in agreement with the model simulations. For instance, a closer look at the summary SAI of the simulations for these stimuli (see Fig 3) reveals a steep increase in the height of the first peak for the ramped sound which indicates a specific carrier salience extraction. The simulations performed with the stimuli with longer *T*_1/2_ (i.e. 16 ms and 32 ms) showed that damped sounds also elicit an increase of the first peak height.

**Fig 10:**
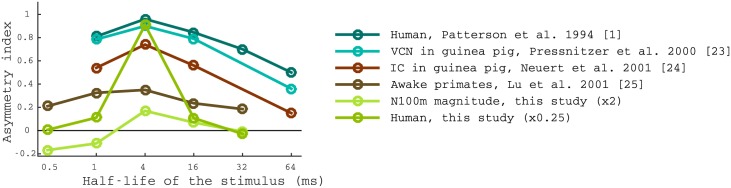
Comparison between our results and previously reported measures of the perceptual asymmetry between ramped and damped sinusoids. Comparison between asymmetry preference of ventral cochlear nucleus [23], inferior colliculus [24], cortical neurons [25], human psychophysical performance in discriminating the ramped and damped sinusoids in A1 [1], the N100m magnitude temporal asymmetry, and psychophysical perceptual asymmetry measured in this work. Multiplicative factors (2 and 0.25, respectively) were applied to rescale the results of our study in order to improve visualisation. Note that the absolute values of the indices depend on the individual scale of each quantity.

We observed that ramped stimuli are associated with a stronger tonal percept, particularly for half life times of *T*_1/2_ = 1—16ms, in agreement with the landmark study from Patterson and colleagues [14]. Moreover, we found that the maximal asymmetry between the perceived salience of ramped and damped stimuli occurs at *T*_1/2_ = 4ms (see Fig 4a). This result is fully in line with previous studies on perceptual asymmetry as shown in Fig 10.

Temporal asymmetry indices for the rest of *T*_1/2_ values vary across studies. There are two potential reasons for such a variability. First, the N100m amplitude is larger for *T*_1/2_ (e.g., 0.5 ms and 1 ms stimuli) and the transient often does not reach a sharp maximum, which hampers the identification of the minimum. Speculatively, this variability in the N100m may underlie part of the perceptual variability. Second, the tonal sensation of stimuli with short *T*_1/2_ stimuli is very weak due to the presence of the simultaneous *drumming* sensation that occludes pitch. This might explain also the different shape of the psychometric curve obtained in [1].

Responses to ramped and damped sinusoids modulated with a 4 ms envelope’s time constant are significantly different between hemispheres. Specifically, responses to ramped and damped stimuli were largely different in the right hemisphere, but statistically indistinguishable in the left hemisphere (see Fig 6). Moreover, hemispheric differences were not observed in the N100m evoked by any of the remaining 9 conditions. This finding indicates a lateralisation of the mechanisms responsible for temporal asymmetry processing at time scales of about 4 ms.

Time-scale specific hemispheric specialisation has been reported before in connection to language [60] and is the target of the *asymmetric sampling in time* (AST) theory [61]. Based on a large amount of experimental evidence on previous literature, AST assumes that the right hemisphere responds preferably to processes requiring longer time scales, whilst the left hemisphere responds preferably to short modulations. However, further investigations are needed to investigate the specific relationship of temporal integration processes and the AST model. Our robust finding of an asymmetry for sounds with 4 ms half-life time indicates that related sounds with slightly different envelopes and durations might be used to further specify auditory processing of the left and right hemisphere.

Our modelling results further suggest that the N100m is related to pitch decoding, as frequently reported in the literature (e.g. [27]). In this work we emphasised that the adaptive processing as implemented in both models is a key to understand the perception of asymmetric sounds and the observed differences in the N100m morphology. Although the spectral analysis on the basilar membrane and the neural transduction process enhance temporal asymmetry to a certain extent [15], this enhancement is indeed not sufficient to explain perceptual effects [15].

Autocorrelation models [11, 13] have been shown to be very successful in pitch extraction of complex tones, but stimulus-dependent temporal integration was required to explain how the auditory system furnishes the balance between temporal resolution and robust pattern recognition [17].

In contrast, the two idealised computational models considered in this study were able to amplify this temporal asymmetry and successfully predict the perceived differences between ramped and damped stimuli (see Fig 4b and 4d). Furthermore, the top-down model accurately predicted the magnitude of the evoked N100m. This result, robustly cross-validated across a large set of samples, suggests that temporal asymmetry encoding may be also mediated by a hierarchical process with top-down driven stimulus-specific integration windows.

However, a more detailed identification of the biophysical processes underlying such stimulus-dependent temporal integration is out of the scope of this study. Our hypothesis is that pitch integration is drawn on the basis of a harmonic pattern of connectivity in alHG [62]. Another potential contributor to the rapid detection of auditory stimuli is neuromodulation [63], a very recent and interesting hypothesis which has not been analysed yet using non-invasive recordings in human subjects.

In summary, the current study provides further evidence that the N100m magnitude indicates the presence of a neurophysiological mechanism encoding pitch saliency in auditory temporal asymmetry, and suggest that pitch salience asymmetry can only be explained by means of adaptive windows of temporal integration. This process seems to be an important component in the perception of natural communication sounds, whose onsets often exhibit complex temporal and spectral changes within the first milliseconds [36, 37] like the ramped and damped sinusoids.
